# Carbon nanoparticles enhance potassium uptake via upregulating potassium channel expression and imitating biological ion channels in BY-2 cells

**DOI:** 10.1186/s12951-020-0581-0

**Published:** 2020-01-28

**Authors:** Lijuan Chen, Jinchu Yang, Xiang Li, Taibo Liang, Cong Nie, Fuwei Xie, Kejian Liu, Xiaojun Peng, Jianping Xie

**Affiliations:** 1 Key Laboratory of Separation Science for Analytical Chemistry, Dalian Institute of Chemical Physics, Chinese Academy of Sciences, Dalian, 116023 China; 2 University of Chinese Academy of Sciences, Beijing, 100049 China; 3 State Key Laboratory of Fine Chemicals, Dalian University of Technology, Dalian, 116024 China; 4 Technology Center, China Tobacco Henan Industrial Co. Ltd, Zhengzhou, 450000 China; 5 Zhengzhou Tobacco Research Institute of CNTC, Zhengzhou, 450001 China

**Keywords:** Carbon nanoparticles, Potassium uptake, Gene expression, Ion channels

## Abstract

**Background:**

Carbon nanoparticles (CNPs) have been reported to boost plant growth, while the mechanism that CNPs enhanced potassium uptake for plant growth has not been reported so far.

**Results:**

In this study, the function that CNPs promoted potassium uptake in BY-2 cells was established and the potassium accumulated in cells had a significant correlation with the fresh biomass of BY-2 cells. The K^+^ accumulation in cells increased with the increasing concentration of CNPs. The K^+^ influx reached high level after treatment with CNPs and was significantly higher than that of the control group and the negative group treated with K^+^ channels blocker, tetraethylammonium chloride (TEA^+^). The K^+^ accumulation was not reduced in the presence of CNPs inhibitors. In the presence of potassium channel blocker TEA^+^ or CNPs inhibitors, the *NKT1* gene expression was changed compared with the control group. The CNPs were found to preferentially transport K^+^ than other cations determined by rectification of ion current assay (RIC) in a conical nanocapillary.

**Conclusions:**

These results indicated that CNPs upregulated potassium gene expression to enhance K^+^ accumulation in BY-2 cells. Moreover, it was speculated that the CNPs simulated protein of ion channels via bulk of carboxyl for K^+^ permeating. These findings will provide support for improving plant growth by carbon nanoparticles.

## Background

Potassium is an essential macronutrient for all plants growth [1–3], which is abundant in plant cells and required for multiple functions, such as maintenance of osmotic pressure, activation of numerous enzymes, and enhancement of photosynthesis. It is well established that the K^+^ uptake is characterized by biphasic uptake kinetics, a high-affinity transport system (mechanism 1, i.e., transporters) and a low-affinity transport system (mechanism 2, i.e., K^+^ channel) [4]. At low external concentrations of K^+^ (≤ 0.2 mM), the mechanism 1 works against an electrochemical gradient with consumption of energy. While at high external concentrations of K^+^ (≥ 0.2 mM), the mechanism 2 operates mostly via the activity of channels without energy.

For the past few years, there is growing interests for researchers in promoting the crop production with carbon nanomaterials [5, 6]. Carbon nanomaterials were useful in seed germination [7], root growth [8] and photosynthesis [8, 9]. Carbon nanotubes can stimulate growth of tobacco cells, by promoting expression of gene and aquaporin [10]. Low dose of multi-walled carbon nanotubes (MWCNTs) was proved to be beneficial in improving water absorption, increasing of plant biomass and the uptake of essential nutrients, such as Ca, Fe [11]. Potassium plays an important role in plant growth. In previous reports, researchers mainly focus on changes of plant biomass, while rarely on the effect that nanomaterials exerted on potassium accumulation in plants. Therefore, uncovering the effect of nanomaterials on potassium accumulation in plants will better clarify the mechanism of carbon nanomaterials for promoting plant growth.

Whether carbon nanomaterials can facilitate K^+^ uptake in biological system, researchers hold quite the opposite opinions. Some researchers believe that carbon nanomaterials suppress the activity of potassium channel and block K^+^ uptake in mammalian cells [12–15]. For instance, it was found that C_60_ neat fullerenes hindered the function of K^+^ channels by binding with K^+^ channel proteins via computer simulation methods [16]. Similarly, it was reported that MWCNTs inhibited potassium channel activities in PC12 cells and CHO cells via current decrease in the whole-cell electrophysiological recording [17]. However, others insist that carbon nanomaterials can enhance K^+^ uptake in biological system, although reports holding this view are limited so far. For example, graphene oxide (GO) was found to be internalized into primary astrocytes and could upregulate inward-rectifying K^+^ channels and Na^+^-dependent glutamate uptake [18]. Single-Walled Carbon Nanotubes (SWCNTs) mimic potassium channels for K^+^ uptake by biomimetic devices [19]. Nevertheless, the effect of carbon nanomaterials on K^+^ uptake was conflicting due to the diverse and stable nanostructures. Carbon nanotubes had a cylindrical channel with a diameter of 1 nm formed with the graphene sheet, and the carbonyl on the entrance of channel preferentially permitted K^+^ to pass through. While C_60_ fullerene with spherical structure, and an average diameter of 0.72 nm, may fill in the ion channels and prevent K^+^ from entering cells [12]. Whereas, there are rare reports about carbon nanomaterials enhancing K^+^ uptake in plant cells.

BY-2 cells are used for research of K^+^ uptake, due to their rapid growth and being synchronized, especially requirement of K^+^ for growth [20, 21]. In addition, Shaker-like K^+^ channel genes in BY-2 cells were confirmed and named *NKT1*, *NtKC1* and *NTORK1*. The inward rectifying *NKT1* channel was involved in cell division through uptake of K^+^ into cells, and the outward rectifying K^+^ channel *NTORK1* activity was concerned with cell cycle in *Xenopus oocytes *[20, 21]. The transporter gene *NtHAK1* was involved in K^+^ uptake under low K^+^ conditions.

**Fig. 1:**
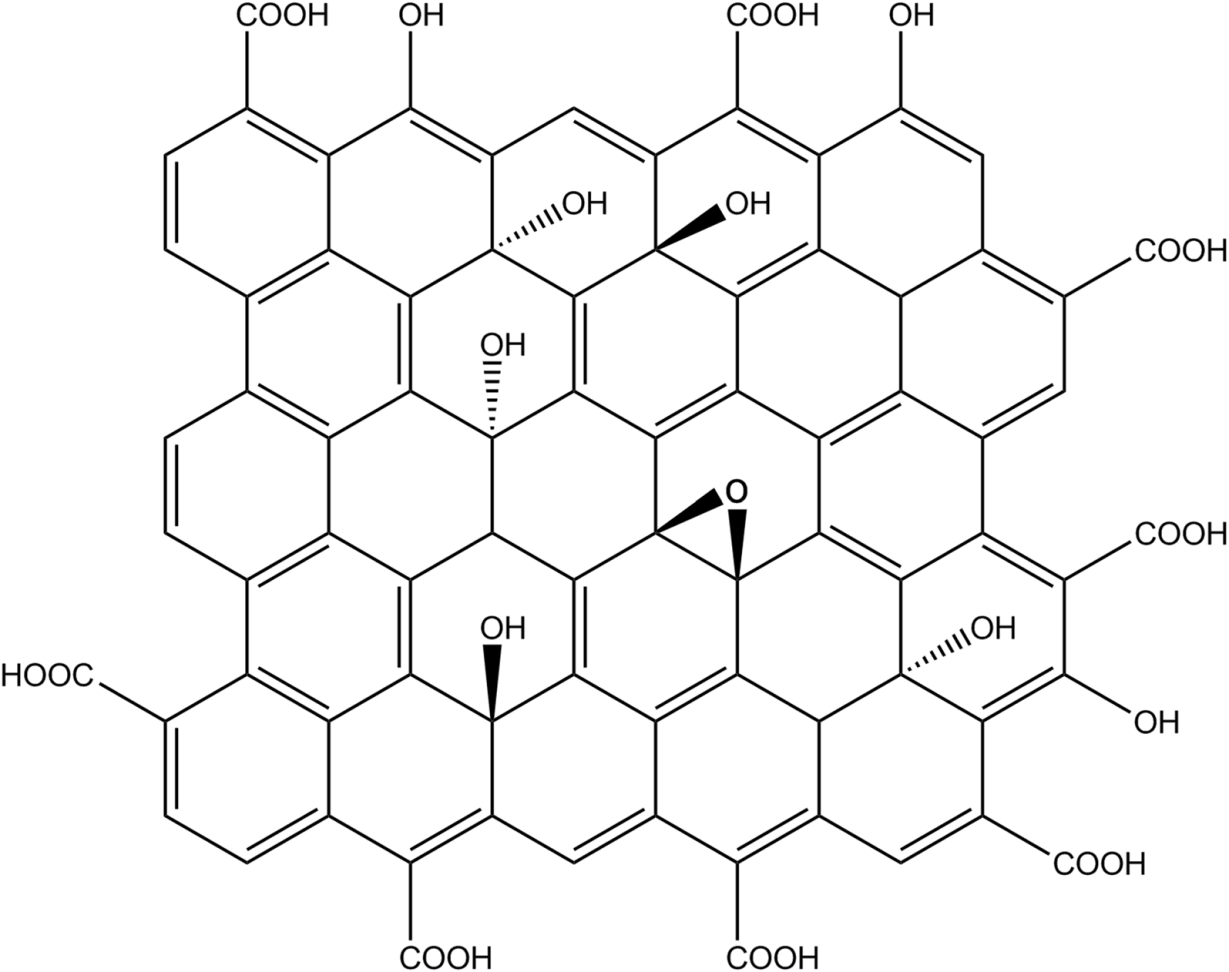
The proposed molecular structure of CNPs

In this study, we employed BY-2 cell for nanobiotechnology research to elucidate the mechanisms that CNPs promoted accumulation of K^+^. The proposed molecular structure of CNPs (Fig. 1) was shown according to previous characterization [22, 23]. BY-2 cells were incubated with different concentrations of CNPs. It was hypothesized that the genes involved in K^+^ uptake were modulated by CNPs to increase the activities of K^+^ influx channels. The change of gene expression and K^+^ channel activity in BY-2 cells was measured. The ion selectivity of CNPs was also evaluated by RIC measurement. The effect of CNPs on plant cell performance was assessed, including changes in intracellular amino acid content and pH value of extracellular matrix. Here, we demonstrated that the concerned K^+^ uptake genes were upregulated by CNPs. In addition, we deduced that CNPs embed on the membrane and acted as biological ion channels for K^+^ permeating. These findings could provide explanation for carbon based nanomaterials in promoting plant growth.

## Methods

### Material

Tobacco BY-2 (*Nicotiana tabacum* L. cv. Bright Yellow 2) suspension cells were cultured with Murashige and Skoog (MS) liquid medium supplemented with sucrose (30 g/L) and 2,4-dichlorophenoxyacetic (2,4-d, 1 mg/L). The suspension cells were sub-cultured at 4-day intervals by transferring 20 mL of suspension into 40 mL of fresh medium, and were shaked under 133 rpm at 27.5 °C in the dark. CNPs were prepared as described in Chen et al. [22] and the preparation process was as follows. Two high purity graphite plates (99.999%) were immersed in deionized water containing 0.1% ethylene glycol, and then voltage was applied on the edge of the graphite plates. The voltage was set at 16 V and current of 0.2 A was employed on graphite electrodes for a few days until the solution became black at room temperature.

### Determination of cell biomass and K^+^ content in cells

Suspension cells were filtered from the MS liquid medium by cell strainer (40 μm, Falcon). Then the cells were washed three times to remove the extracellular K^+^ with 3% sucrose solution. After centrifugation of cells at 200×*g* for 3 min, the fresh biomass of cells was recorded. Then the BY-2 cells were resuspended in ultrapure water. For K^+^ extraction, cells were broken by ultrasonication (JY92-2D, Scientz, China). The supernatant was collected, and K^+^ concentration was determined using inductively coupled plasma mass spectrometer (ICP-MS, Agilent 7500a, USA).

### TGA-FTIR analysis of CNPs

The pyrolysis of CNPs and CNPs treated with ethanol was determined using thermogravimetric analysis (TGA) coupled to a Fourier transform infrared spectrum (FTIR) technique. The 8 mg sample powder was placed on small PtHT crucibles. The TGA pyrolysis program was operated under a nitrogen atmosphere (100 mL/min). The sample was heated up to 100°C and equilibrated for 20 min to remove H_2_O and air. The heating rate was set to 15 °C/min from 100 °C up to the final temperature of 800 °C. The generated gas phase volatiles were transferred via a heated line (230 °C) interfaced to the FTIR.

### K^+^ uptake related gene expression

Total RNA of BY-2 cell was extracted and purified using plant total RNA isolation kit (Sangon Biotech, China). Complementary DNA was synthesized using M-MLV reverse transcriptase (Takara) with oligo (dT) primers. These K^+^ uptake related gene primers were synthetized according to the National Center for Biotechnology Information database (NCBI), including the inward-rectifying K^+^ channel *NKT1* (GenBank: AB196790) and *NtKC1* (GenBank: AB196791), outward-rectifying K^+^ channel *NTORK1* (GenBank: AB196792), plasma membrane H^+^-ATPase *NHA1* (GenBank: AY383599) and K^+^ transporter *NtHAK1* (GenBank: DQ841950). Real-time quantitative PCR was performed on a CFX96 real-time system (Bio-RAD, USA) using LightCycler® 480 SYBR Green I Master (Roche) according to the manufacturer’s instructions. The relative transcript level of genes was quantified by 2^−∆∆CT^ method [24]. The control gene (*Actin*) was used as normalization for the test gene transcript.

### Non-invasive micro-test technology (NMT) for potassium ion flux analysis

Net fluxes of K^+^ were measured using NMT system (Younger USA LLC, Amherst, MA01002, USA) [25, 26]. BY-2 cells were incubated with bath solution (46.88 mM KCl, 0.1 mM CaCl_2_, 0.3 mM Mes, pH 5.9 unbuffered) in petri dish. The work microelectrode was positioned in close proximity to a selected BY-2 cell and left to equilibrate for a few minutes. The micropipettes were filled in selective liquid ion-exchange cocktails with a length of 180 μm (K^+^ LIX, XY-SJ-K, Younger, USA). The microelectrode was stepped from one position to close to the selected cell in a predefined sampling routine. Pre-pulled and silanized microsensor (Φ1.5 ± 0.5 μm, XY-CGQ-02, Younger USA) was filled with a backfilling solution (100 mM KCl). An Ag/AgCl wire microsensor holder YG003-Y11 (Younger, USA), was used as the reference microsensor. The exported raw data of micro-volt differences (△μV) was then imported and converted into net K^+^ fluxes using the JCal V3.3 (a free MS Excel spreadsheet, youngerusa.com or xbi.org).

### Rectification of ion current measurements

Glass capillaries were pulled by Sutter 97 to prepare conical nanocapillaries. Two Ag/AgCl electrodes were used to record a current–voltage curve via a patch-clamp amplifier [27]. One electrode is placed in the reservoir, facing the nanocapillary. The other one was inserted into the capillary. The conical nanocapillaries were filled with CNPs solution with pure water as a control and GO as a positive control. Transport properties of nanocapillary were measured in KCl, NaCl, NH_4_Cl and CaCl_2_ solution at the concentration of 100 mM.

### Free amino acids analysis

The cell lysis supernatant obtained from ultrasonication was also collected for amino acids analysis using amino acid analyzer (Biochrom 30 + , Biochrom Ltd, UK). The Li buffer was added to supernatant for adjustment of the pH value. Amino Acid Standard (Sigma) was used to quantify the concentration of amino acid in lysis supernatant.

### Measurement of extracellular matrix pH value

Cells were incubated with varied concentration of CNPs for 12 h. Extracellular matrix was separated from cells by cell strainer and collected for determination of pH value.

## Results and discussion

### K^+^ uptake in BY-2 cells

**Fig. 2:**
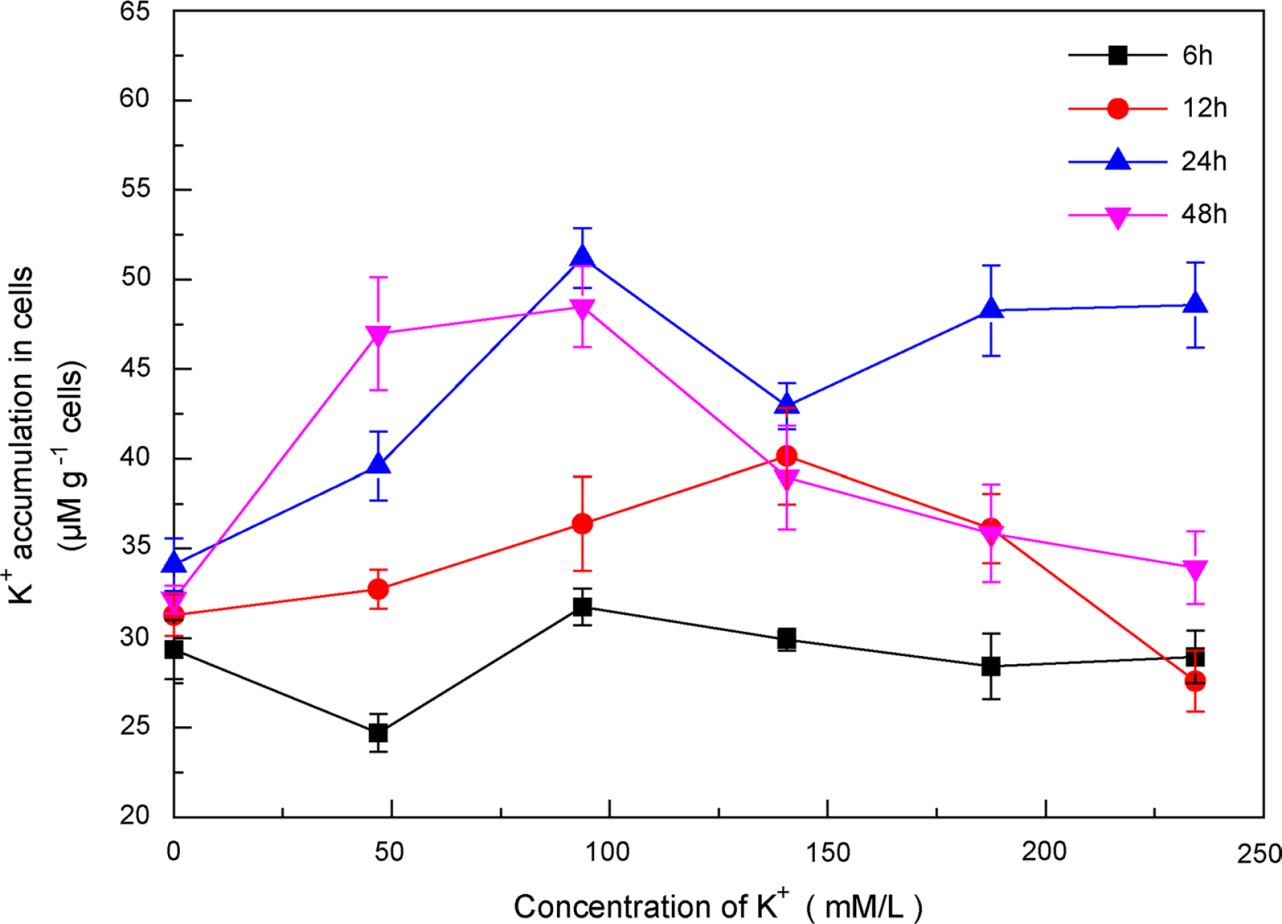
Effect of addition K^+^ concentration and incubation time on the intracellular K^+^ accumulation in BY-2 cells. Error bars indicate the standard error of the mean (n = 4)

The K^+^ uptake in BY-2 cells was investigated. As shown in Fig. 2, intracellular accumulated K^+^ content increased with the increasing concentration of KCl and reached a relative maximum value when the concentration of K^+^ was 93.75 mM/L. Then intracellular K^+^ accumulation no longer increased. Intracellular K^+^ accumulation was also related to the incubation time of KCl. The intracellular K^+^ accumulation increased with the prolonging of the incubation time, and was significantly different compared with control group when concentration of K^+^ was lower than 93.75 mM/L. The intracellular K^+^ accumulation increased with the addition of increasing concentration of K^+^, which was similar with K^+^ uptake in barley roots from 0.2 to 50 mM/L of KCl [4]. In previous publications, K^+^ accumulation in plant cells (BY-2 cells) reached a threshold and then initiated the next cell cycle. Division cycle of BY-2 cells is about 12 h [21], which explained that higher intracellular K^+^ was accumulated when the incubation time was multiples of division cycle.

**Fig. 3:**
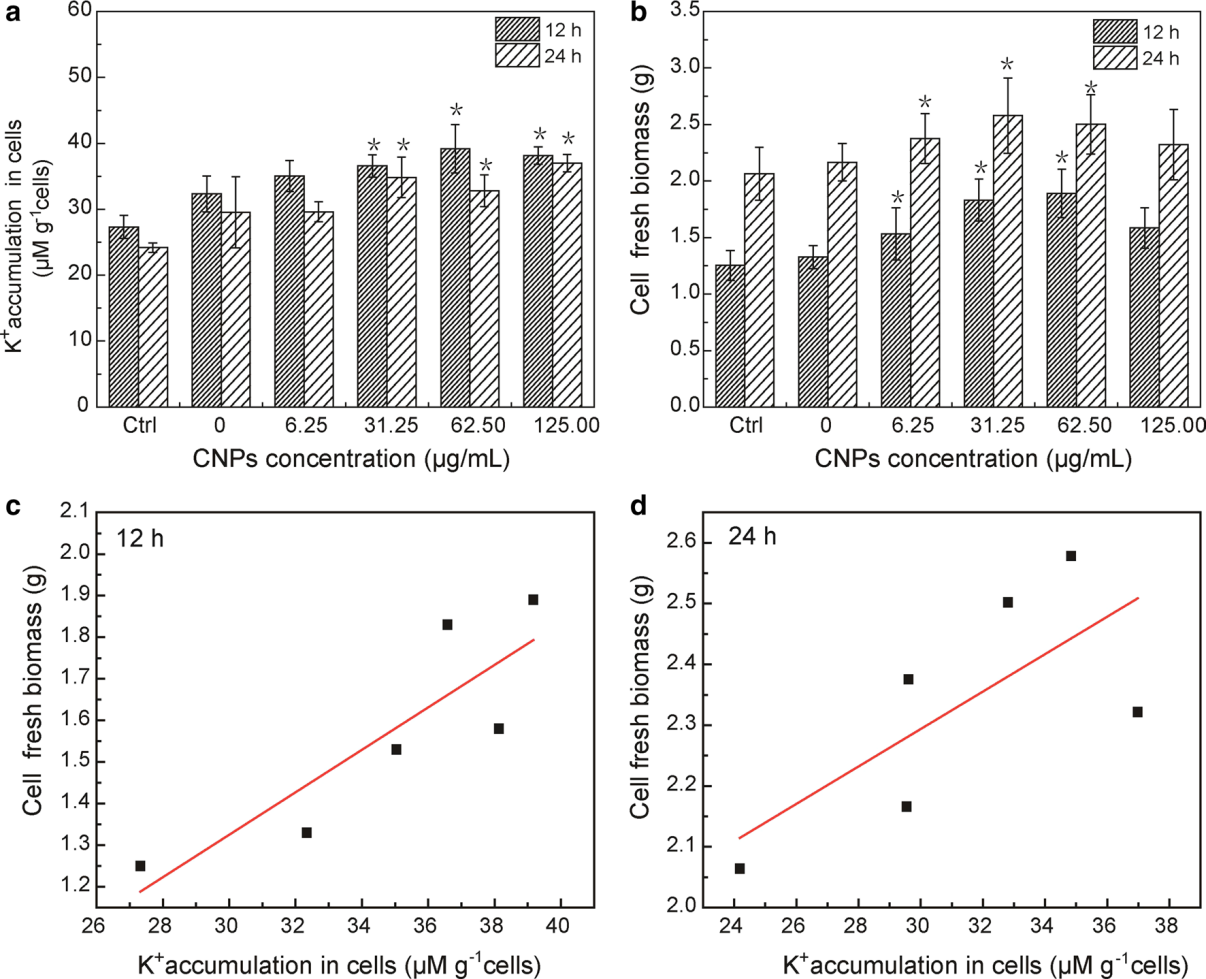
Effect of CNPs concentration and incubation time on the intracellular K^+^ accumulation in BY-2 cells (**a**) and BY-2 cell fresh biomass (**b**). K^+^ content was measured in medium containing 46.88 mM K^+^, except for control group. Error bars indicate the standard error of the mean (n = 4). Asterisk (*) indicates significant difference compared with control group and group containing 46.88 mM K^+^ without CNPs (p < 0.05). **c**, **d** Relationship between cell fresh biomass and K^+^ accumulation in cells using an Bivariate statistical model with cell fresh biomass as Y variable. **c** The Bivariate model is highly significant (p < 0.026, R^2^ = 0.865). **d** The Bivariate model is no obvious significant (p > 0.05, R^2^ = 0.718)

In Fig. 3a, with different concentration of CNPs and a fixed concentration of K^+^(46.88 mM/L) in BY-2 cells culture medium, K^+^ accumulation in cells increased with the increasing concentration of CNPs. When the concentration of CNPs reached 62.50 μg/mL, intracellular K^+^ accumulation was almost 40 μΜ/g cells and increasing by 21.12% compared with the control group (with 46.88 mM/L KCl and without CNPs). There was no obvious difference in K^+^ accumulation in cells when the incubation time was 12 h and 24 h. The cell fresh biomass increased with addition of CNPs (Fig. 3b). As shown in Fig. 3c, cell fresh biomass exhibited correlation with K^+^ accumulation in cells (12 h). As shown in Additional file 1: Fig. S1A, CNPs did not affect the intracellular K^+^ accumulation under the condition without addition of K^+^. The intracellular K^+^ accumulation displayed significantly difference when the concentration of CNPs reached 125.00 μg/mL and the cells were incubated in 20 mM/L of KCl for 12 h and 24 h (Additional file 1: Figure. S1B). These results indicated that CNPs exerted influence in the presence of a certain concentration of K^+^.

**Fig. 4:**
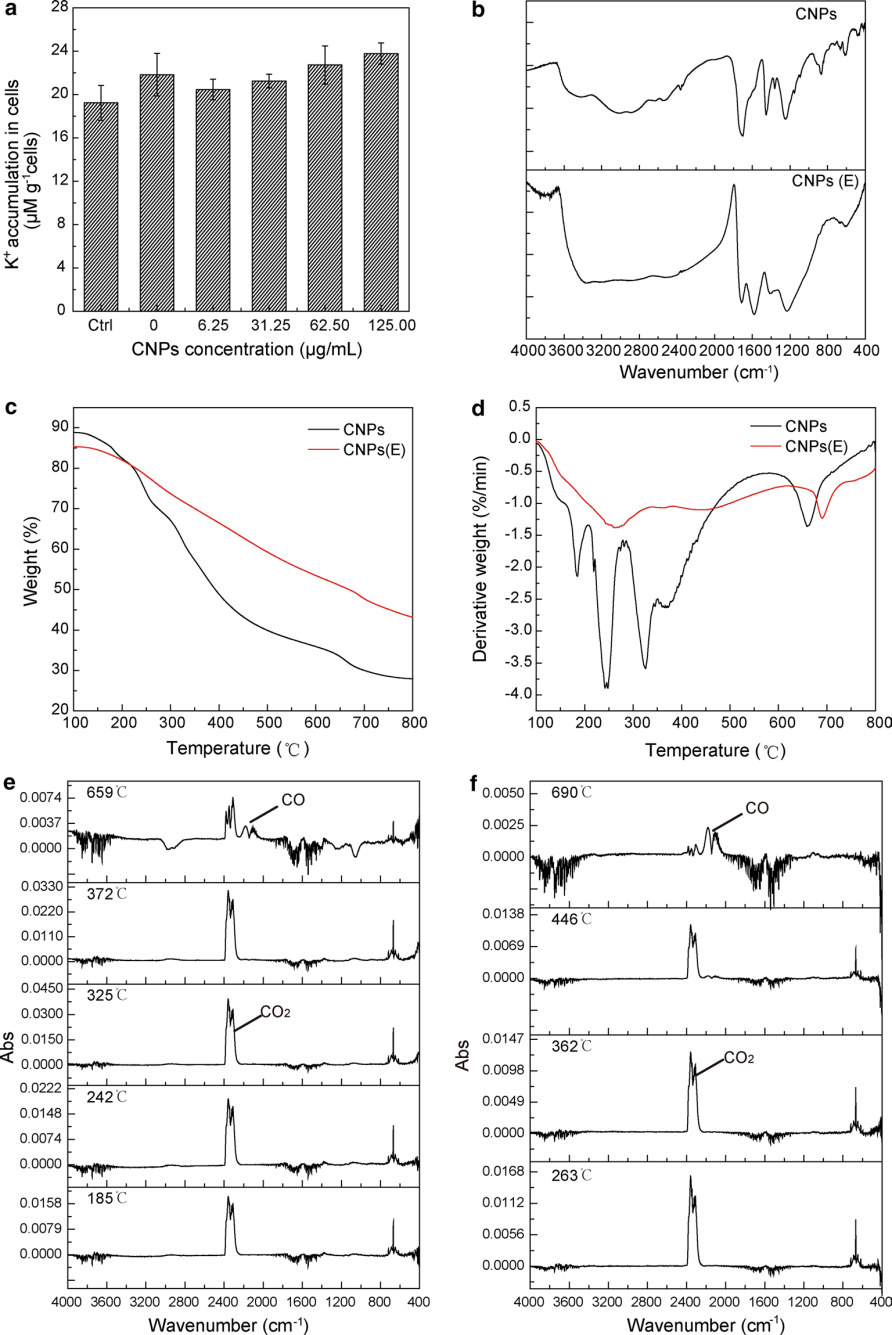
**a** Effect of CNPs treated by ethanol on the intracellular K^+^ accumulation in BY-2 cells. K^+^ accumulation was measured in medium containing 46.88 mM K^+^, except for control group. Error bars indicate the standard error of the mean (n = 4). **b** FT-IR spectra of CNPs and CNPs(E). **c**, **d** TG and DTG thermograms for CNPs and CNPs(E). **e**, **f** FTIR spectra representing absorbance at different temperatures of CNPs and CNPs(E). **e** CNPs, **f** CNPs(E). *CNPs(E)* CNPs treated by ethanol

The CNPs treated with ethanol did not promote K^+^ accumulation in BY-2 cells in Fig. 4a (ethanol was removed from CNPs). When ethanol and CNPs solution was mixed with a proportion of 2:1 (v:v), the CNPs aggregated to form larger particles and precipitated in the mixed solution. While the CNPs were resuspended in the pure water after removal of ethanol, and the dispersibility became worse compared with the original CNPs. In the IR spectra of CNPs, the broad peak located at 3200–2500 cm^−1^ was assigned to dimer carboxyl. When CNPs were treated with ethanol, the carboxyl characteristic peaks appeared to be wide, ranging from 3200 to 1900 cm^−1^. Besides, the intensity of C=O and the C=C in aromatic ring (the common feature of graphitic sp^2^ hybridization carbons) located at 1680 and 1650–1450 cm^−1^ was changed compared with that of CNPs (Fig. 4b). The differences between CNPs and CNPs treated with ethanol also were assessed by a thermogravimetric analysis (TGA) coupled to a Fourier transform infrared spectrum (FTIR). TGA and DTG thermograms of the pyrolysis displayed obvious difference in CNPs and CNPs treated with ethanol in Fig. 4c, d. There is a higher weight loss rate and more pyrolysis stages in CNPs than that in CNPs treated with ethanol. In the FTIR spectra (Fig. 4e, f), the formation of CO_2_ at different stages originated from the fracture and recombination of carbonyl and carboxyl group (corresponding to different distribution, as shown in Fig. 1), while the generated CO is derived from the rupture of carbonyl and ether bond [28]. The carbonyl and carboxyl group on CNPs was disrupted by ethanol from the TGA-FTIR analysis. Therefore, it was speculated that the carboxyl groups were responsible for facilitating K^+^ uptake in BY-2 cells.

### Expression of K^+^ uptake related genes in BY-2 cells

**Fig. 5:**
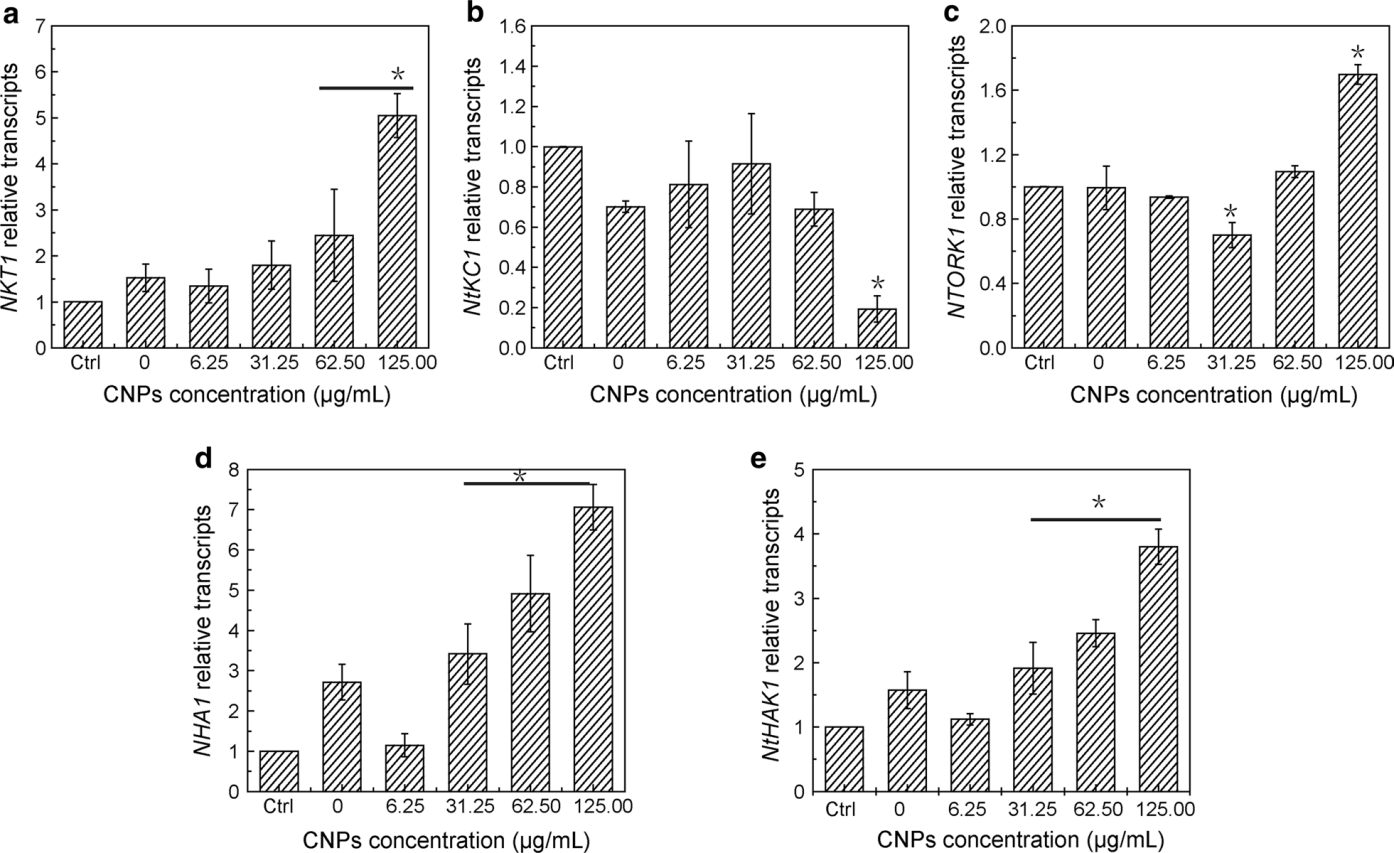
Gene expression analysis of K^+^ channels (**a**–**c**), plasma membrane H^+^-ATPase (**d**) and K^+^ transporter (**e**) treated with CNPs in BY-2 cells. Gene transcripts level was measured in medium containing 46.88 mM K^+^, except for control group, and the CNPs exposure time was 12 h. Data represented the mean and SE (n = 3). Asterisk (*) indicates significant difference compared with control group and group containing 46.88 mM K^+^ without CNPs (p < 0.05)

Inward-rectifier *NKT1*, *NtKC1* and outward-rectifier *NTORK1* was involved in K^+^ uptake identified in BY-2 cells. *NKT1* and *NTORK1* gene expression was upregulated in a linear manner with the increasing CNPs concentration, while *NtKC1* gene expression was downregulated (Fig. 5a, c). The plasma membrane H^+^-ATPase *NHA1* and K^+^ transporter *NtHAK1* was also upregulated (Fig. 5d, e). Results of quantitative PCR demonstrated that *NKT1*, *NHA1* and *NtHAK1* relative transcripts exhibited significant difference and were at least twofold higher compared with control and other treated groups at a concentration of 62.50 and 125.00 μg/mL. Results of gene expression were basically in agreement with the results of K^+^ accumulation in cells. These results indicated that *NKT1* channels, *NHA1* and *NtHAK1* played an important role in CNPs promoting K^+^ uptake. Potassium channel *NKT1* exert high-capacity K^+^ uptake in BY-2 cells, which represents a low-affinity methanism for K^+^ uptake when the external K^+^ concentration was greater than 0.2 mM. However, when the external K^+^ concentration was in the micromolar range from 0.002 to 0.2 mM, high-affinity systems worked, such as K^+^ transporter *NtHAK1*. It has been reported that the K^+^ transporter seemed to be less important for K^+^ uptake in BY-2 cells. The plasma membrane H^+^-ATPase was found to maintain the proton gradient in plants [29] and fungi [30]. Overexpression of H^+^-ATPase *AHA2* promoted hyperpolarization of the plasma membrane in guard cell in response to blue light, and increasingly induced K^+^ uptake into guard cells in *A. thaliana* transgenic plants [31].

### Effect of CNPs on K^+^ flux in BY-2 cells

**Fig. 6:**
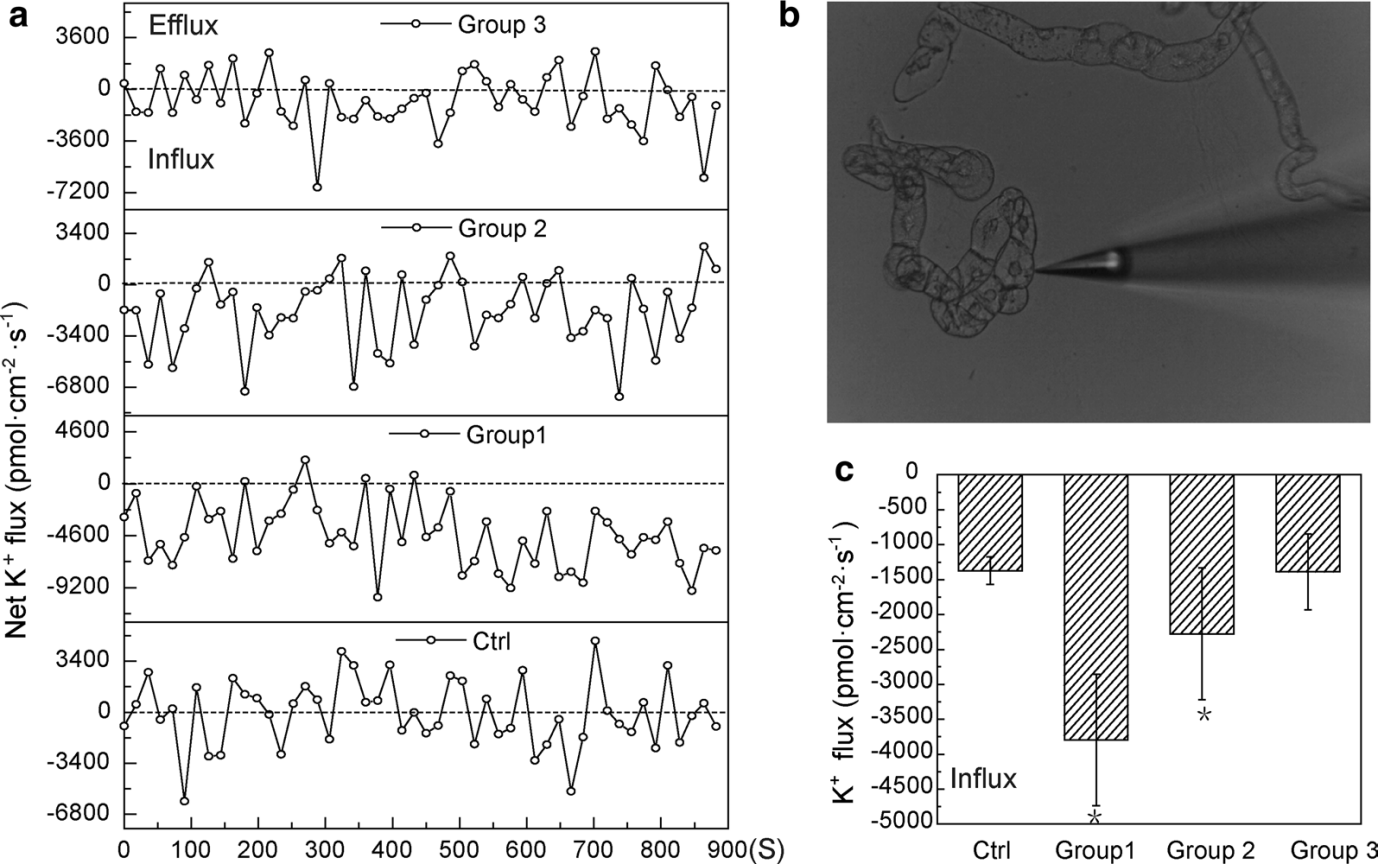
Effects of CNPs and inhibitor treatments on net K^+^ fluxes in BY-2 cells. **a** K^+^ fluxes were determined with after different CNPs treatments lasting for 900 s measurements. **b** Schematic diagram of test sample. **c** Mean value of K^+^ fluxes at different concentration of CNPs in cells. Group 1: 62.50 μg/mL CNPs, Group 2: 125.00 μg/mL CNPs, Group 3: 125.00 μg/mL CNPs + 10 mM TEA^+^. The mean value of each group contains six individual cell, and error bars represent SE, n = 6. Asterisk (*) indicates significant difference compared with control group (p < 0.05)

The net K^+^ flux response to CNPs exposure was measured using the NMT technology to further understand the mechanism of promoting potassium accumulation in BY-2 cells. As shown in Fig. 6a, the K^+^ influx reached high level after treatment with varied concentration of CNPs for 12 h, and was significantly higher than the control group and the negative group treated with K^+^ channels blockers, TEA^+^ (p < 0.05, n = 6). The average K^+^ influxes ranged from the maximum value 3797.50 ± 942.46 to the minimum value 1372.95 ± 196.11 pmol cm^2^ s^−1^. Moreover, the highest value of K^+^ influx almost was a threefold and twofold increase compared with the control and the negative control group, respectively (Fig. 6c). There was no obvious significance between the negative control and control group. The K^+^ influx increasing with CNPs application in NMT assay was probably related with transmembrane ion channels. To verify whether the observed K^+^ was caused by the activity of K^+^ channels, BY-2 was pretreated with 10 mM tetraethylammonium chloride (TEA^+^), a well-known inhibitor of K^+^ channels. After treatment of TEA^+^ for 12 h, K^+^ influx was inhibited in the presence of CNPs. Based on these data, it could be concluded that the net K^+^ influx was mainly originated from the enhanced activity of *NKT1* induced by CNPs.

### Effect of K^+^ channel blockers and CNPs blocker on K^+^ uptake

**Fig. 7:**
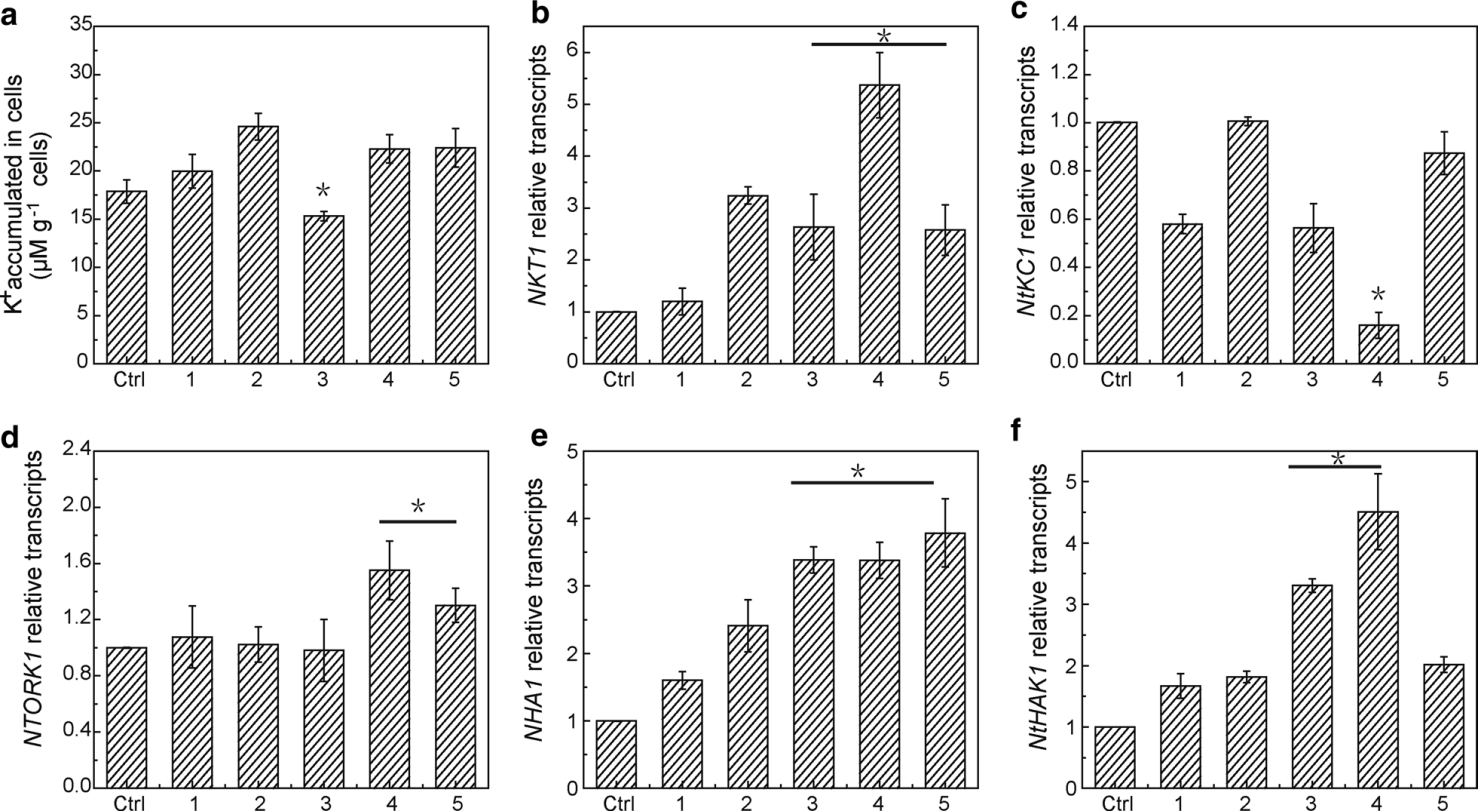
K^+^ accumulation (**a**) and gene expression (**b**–**f**) in BY-2 cells treated with CNPs and inhibitors. Cells were treated with CNPs for 12 h and gene expression was measured by quantitative RT-PCR. Data represent the mean and SE (n = 3). Values of actin relative transcripts were normalized to 1. Asterisk (*) indicates significant difference compared with control group and group containing 46.88 mM K^+^ without CNPs (p < 0.05). 1: 46.88 mM K^+^, 2: 46.88 mM K^+^  + 62.50 μg/mL CNPs, 3: 46.88 mM K^+^  + 62.50 μg/mL CNPs + 10.00 mM TEA^+^, 4: 46.88 mM K^+^  + 62.50 μg/mL CNPs + 0.16 mM genistein, 5: 46.88 mM K^+^  + 62.50 μg/mL CNPs + 0.30 mM methyl-β-cyclodextrin

BY-2 cells further treated with TEA^+^ (10 mM) exhibited a significant decrease in K^+^ accumulation compared with CNPs treated group, while exhibited no significant difference with the control groups (Fig. 7a). The results indicated that K^+^ uptake was inhibited by TEA^+^ to some extent. No obvious effect of K^+^ accumulation was found by addition of genistein or methyl-β-cyclodextrin. Gene expression analysis showed that K^+^ channel blockers and CNPs blockers changed gene transcripts and was not in accord with results of K^+^ accumulation in cells (Fig. 7b–f). Genistein and methyl-β-cyclodextrin were proved to inhibit CNPs entering BY-2 cells, which demonstrated that another pathway of CNPs promoting K^+^ uptake was not via entering BY-2 cells.

### Rectification of ion current in nanopores

**Fig. 8:**
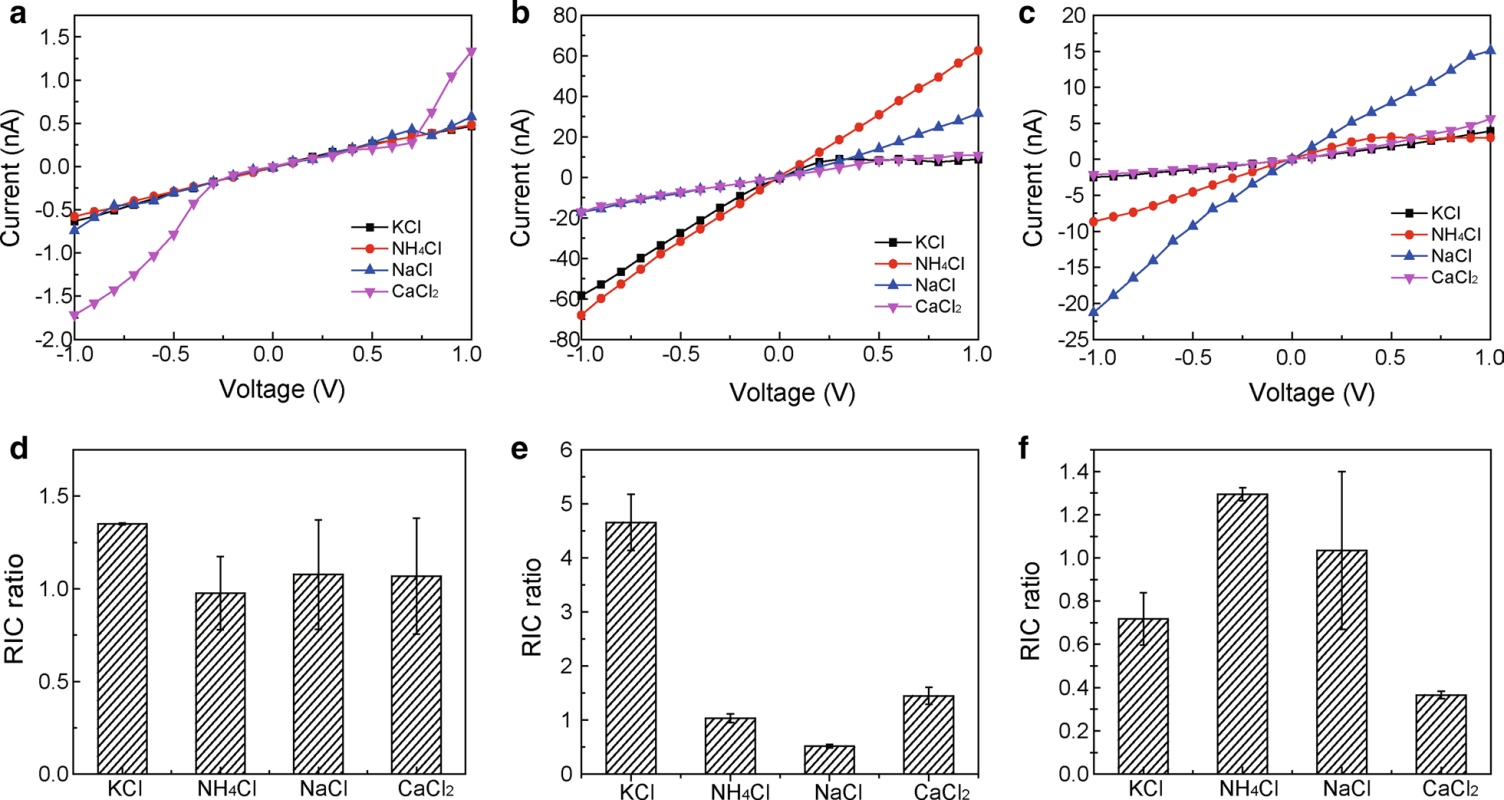
**a**–**c** I–V curves and **e**–**f** ICR ratio of control, CNPs and GO in conical nanopore. **a**, **d** Control group filled with pure water, **b**, **e**: CNPs treated group, **c**, **f**: GO treated group. The tip and base sizes of conical nanopore are around 4.0 nm and 1.2 μm, respectively

Conical nanopores have non-linear rectification property due to their asymmetrical charge or shape, and they were similar to ion channel on the cell membrane. I–V curves showed for the ionic selectivity characteristic of the conical nanopores in the absence or in the presence of CNPs, with GO as a positive control (Fig. 8a–c). The Rectification of Ion Current (RIC) ratio of K^+^ calculated from the non-linear I–V curve is 4.7 times higher than that of other cations in the presence of CNPs (Fig. 8e). Conical nanopore exhibited high selectivity towards NH_4_^+^ when was filled with GO (Fig. 8f). Moreover, conical nanopores exhibited no selectivity for the monovalent cation and divalent cation when were infused with pure water (Fig. 8d). The substantial difference for the ion selectivity may be attributed to the chemical characteristics of CNPs or GO, neglecting the conical nanopore shapes in these cases. Therefore, it was speculated that carboxyl groups of CNPs may imitate the potassium channels for K^+^ uptake when CNPs were adsorbed on the membrane of BY-2 cells.

The structure of ion channels provides guidance for designing of biomimetic nanopores. Charged carboxyl of amino acid side chains played a vital role in the selectivity properties of biological ion channels. Carbon nanotubes have been used for biomimetic nanopores research due to the negatively charged carboxyl. SWCNTs were used for the ion conductance and found to be mimics of ion channels for K^+^ permeating [19]. These negatively charged carboxylates at the entrance of the SWCNTs exerted enormous impact on the ion transport, especially on K^+^ permeability. Moreover, SWCNTs have been designed as biomimetic nanopores for ion selectivity based on voltage-gated ion channels [32]. Though there are carboxyl groups on the GO, GO did not exhibit high K^+^ selectivity due to the distribution of carboxyl groups. In addition, single graphene nanopore loaded on PET membrane (G/PET nanopore) with high ionic flux and high ion selectivity was confirmed in the G/PET nanopore system [33].

### Effect of CNPs on amino acid content in BY-2 cells

Metabolites of amino acid were indicator for CNPs improvement of K^+^ uptake in BY-2 cells. The metabolites of 25 kinds of amino acid from BY-2 cells were identified and these results were shown in Table 1. Amino acid content of the CNPs treated groups was significantly different from that of the control group. 12 kinds of amino acid content including l-proline, l-arginine and glycine, increased for more than two-times with the increasing concentration of CNPs, and decreased at the concentration of 125.00 μg/mL. However, the content of l-tyrosine was reduced with the increasing concentration of CNPs. The content of amino acid in BY-2 cells was changed under the stress condition induced by CNPs.

**Table 1:** Free amino acids analysis treated with CNPs in BY-2 cells

Retention time	Amino acid	Ctrl	1	2	3	4	5
		μM/g					
22.332	l-Aspartic acid↑↓	0.267 ± 0.026 c	0.399 ± 0.076 c	0.584 ± 0.020 b	0.762 ± 0.220 a	0.701 ± 0.136 a	0.542 ± 0.150 b
25.499	Hydroxy-l-proline	2.240 ± 0.395 b	1.904 ± 0.446 c	2.646 ± 0.209 a	1.450 ± 0.587 d	2.559 ± 0.558 a	1.787 ± 0.101 c
29.211	l-Threonine↑↓	0.869 ± 0.047 d	1.075 ± 0.056 d	1.334 ± 0.091 c	1.750 ± 0.205 b	1.993 ± 0.328 a	1.412 ± 0.078 c
31.352	l-Serine↑↓	1.917 ± 0.034 d	1.919 ± 0.242 d	2.473 ± 0.163 c	3.013 ± 0.235 a	2.742 ± 0.714 b	2.070 ± 0.266 e
36.416	l-Asparagine	1.204 ± 0.097 c	1.313 ± 0.137 c	1.830 ± 0.126 b	2.125 ± 0.066 a	1.897 ± 0.431 b	1.917 ± 0.880 b
38.967	l-Glutamic acid	0.217 ± 0.070 c	0.130 ± 0.042 d	0.161 ± 0.024 d	0.439 ± 0.242 b	0.535 ± 0.040 a	0.247 ± 0.129 c
41.108	Glutamine	1.076 ± 0.137 c	0.983 ± 0.293 c	1.373 ± 0.032 b	1.757 ± 0.206 a	1.174 ± 0.409 c	1.746 ± 0.147 a
50.115	l-Proline↑↓	0.360 ± 0.054 e	0.603 ± 0.56 d	1.099 ± 0.115 c	1.612 ± 0.195 a	1.740 ± 0.341 a	1.408 ± 0.268 b
51.567	Glycine↑↓	1.345 ± 0.183 c	1.304 ± 0.217 c	1.690 ± 0.208 b	2.186 ± 0.213 a	2.210 ± 0.416 a	1.651 ± 0.070 b
53.265	l-Alanine	12.522 ± 1.002 a	11.686 ± 1.326 b	11.296 ± 0.313 b	12.703 ± 0.721 a	9.966 ± 1.176 c	6.456 ± 0.258 d
60.425	l-Valine	2.128 ± 0.098 c	2.280 ± 0.249 c	2.850 ± 0.124 b	3.494 ± 0.182a	3.629 ± 0.661 a	2.341 ± 0.313 c
68.309	L-Methionine↑↓	0.326 ± 0.075 e	0.496 ± 0.050 e	0.664 ± 0.021 d	0.767 ± 0.048 b	0.802 ± 0.206 a	0.710 ± 0.192 c
70.909	Cystathionine↑↓	0.904 ± 0.056 f	1.284 ± 0.050 e	1.663 ± 0.114 d	2.362 ± 0.364 b	2.697 ± 0.513 a	1.882 ± 0.085 c
72.709	l-Isoleucine↑↓	2.134 ± 0.083 e	2.625 ± 0.096 d	3.486 ± 0.214 b	3.954 ± 0.236 a	4.152 ± 0.862 a	3.185 ± 0.345 c
73.825	l-Leucine	0.126 ± 0.076 a	0.128 ± 0.049 a	0.130 ± 0.022 a	0.074 ± 0.012 c	0.062 ± 0.014 c	0.096 ± 0.003 b
77.824	*N*-Leucine↑↓	0.462 ± 0.057 e	0.565 ± 0.009 d	0.664 ± 0.065 c	0.731 ± 0.031 b	0.848 ± 0.145 a	0.679 ± 0.137 c
79.939	l-Tyrosine↓	0.552 ± 0.165 a	0.498 ± 0.168 b	0.514 ± 0.045 b	0.331 ± 0.007 c	0.342 ± 0.110 c	0.300 ± 0.111 c
82.163	β-Alanine↑↓	0.694 ± 0.047 e	0.932 ± 0.070 d	1.297 ± 0.101 c	1.384 ± 0.094 b	1.560 ± 0.221 a	1.236 ± 0.246 c
92.164	γ-Amino-*n*-butyric acid	8.992 ± 0.111 d	8.365 ± 1.015 e	9.282 ± 0.472 c	10.189 ± 0.539 b	11.736 ± 0.282 a	5.954 ± 0.238 f
104.221	l-Lysine	0.842 ± 0.049 e	1.193 ± 0.170 d	1.774 ± 0.193 b	1.563 ± 0.850 c	1.253 ± 0.136 d	2.381 ± 0.208 a
106.940	1-Methyl-l-histidine	0.129 ± 0.023 c	0.150 ± 0.046 b	0.159 ± 0.025 b	0.193 ± 0.034 a	0.062 ± 0.008 d	0.096 ± 0.057 d
108.484	l-Histidine↑↓	0.297 ± 0.020 e	0.385 ± 0.038 d	0.541 ± 0.028 c	0.660 ± 0.036 b	0.731 ± 0.121 a	0.511 ± 0.007 c
112.169	3-Methyl-l-histidine	0.126 ± 0.011 d	0.157 ± 0.038 c	0.257 ± 0.023 b	0.163 ± 0.011 c	0.338 ± 0.062 a	0.229 ± 0.014 b
117.503	l-Carnosine	0.144 ± 0.025 b	0.189 ± 0.043 a	0.145 ± 0.096 b	0.133 ± 0.005 b	0.152 ± 0.042 b	0.103 ± 0.022 c
122.311	l-Arginine↑↓	0.737 ± 0.058 d	1.082 ± 0.178 c	1.545 ± 0.114 b	1.693 ± 0.073 a	1.794 ± 0.390 a	1.539 ± 0.371 b

1:treated with 46.88 mM K^+^

2:treated with 46.88 mM K^+^  + 6.25 μg/mL CNPs

3:treated with 46.88 mM K^+^  + 31.25 μg/mL CNPs

4:treated with 46.88 mM K^+^  + 62.50 μg/mL CNPs

5:treated with46.88 mM K^+^  + 125.00 μg/mL CNPs

↑↓Arrows indicated the content of amino acid increased with the concentration of CNPs, and decreased at concentration of 125.00 μg/mL

The letters “a–e” represent significant difference between groups

Proline adjusted plant to stress response [34]. It has been reported that many plants accumulated proline under stress conditions (drought, low and high temperatures, high salinity) [35, 36]. Proline is not only an osmotic regulator in plant cytoplasm, but also plays an important role in stabilizing the structure of macromolecules, reducing cell acidity, removing ammonia toxicity, and regulating cell redox [37]. Arginine plays an important role in the synthesis of polyamines that acts as the signaling molecule and improves stress resistance during defensive reaction in plants. Its content increased more than two times compared with control group, indicating its involvement in metabolism of BY-2 cells under the CNPs exposure condition. Increase of amino acid content is vital for plants during stress condition, and our results were in agreement with the previous statement. Glycine promoted the uptake of phosphorus and potassium in plants, enhanced plant stress resistance and improved the activity of enzymes. The variation trend of glycine was consistent with that of the K^+^ accumulation in cells promoted by CNPs.

### Effect of CNPs on external medium pH value

The high concentration of CNPs (4 mg/mL) had a low pH value of 2.00. As shown in Additional file 1: Figure S2, incubation of cells with higher concentration of CNPs for 12 h caused obvious change in pH value of culture medium (p < 0.05). Although there was an increase of pH value for the culture medium, all the pH value is still suitable for cell growth. The BY-2 cells may produce secondary metabolites to resist the decrease of pH value. The suitable pH value in culture medium of BY-2 cells is crucial for microbial growth, metabolism, and biosynthesis of secondary metabolites [38].

## Discussions

The intracellular K^+^ accumulation was determined by ICP-MS. It was found that the K^+^ accumulation increased significantly when the CNPs concentration was 61.25 μg/mL compared with that of control group. Subsequently, the K^+^ uptake related genes, including potassium channels and transporter genes, were upregulated in comparison with the control groups analyzed by RT-PCR, especially the inward-rectifying potassium channels gene *NKT1*. CNPs increased K^+^ accumulation in BY-2 cells via upregulating the K^+^ uptake related genes. The change of K^+^ content was basically in agreement with the upregulating genes with the increasing concentration of CNPs. K^+^ entered cells via potassium channels, which belongs to passive transport and was independent of energy. The potassium transport into cells via transmembrane transporters is ATP-dependent. In previous study, it was found that CNPs entered BY-2 cells and aggregated around the cell nucleus, which can explain why CNPs regulated genes expression. In addition, CNPs were also confirmed no effect on the production of ATP [23]. TEA^+^ could significantly inhibit K^+^ uptake in the presence of CNPs, which demonstrated that potassium channels played an important role in K^+^ uptake. Although K^+^ transporter *NtHAK1* gene encoding was upregulated, whose transport activity is dependent on the K^+^ electrochemical gradient across plasma membrane, the contribution of *NtHAK1* transporter to K^+^ accumulated in cells was not significant compared with *NKT1* channels. In the presence of potassium channels blocker TEA^+^, the K^+^ accumulation had no obvious difference with that of the control group. These results elaborated the mechanism that CNPs promoted K^+^ accumulation in cells. H^+^-ATPase *NHA1* pumped H^+^ from the cell cytosol to the extracellular, building up the electrochemical gradient inside and outside of the plasma membrane [39], which was proved less important than *NKT1* for K^+^ uptake.

CNPs exhibited particulate morphology with an average size of 30 nm. There are groups of COOH and OH on the surface of CNPs, and CNPs showed the common feature of graphitic sp^2^ hybridization carbons [22]. The CNPs with carbonyl disturbed by ethanol were proved to have no impact on potassium uptake. Therefore, it was speculated that the carbonyl was crucial for K^+^ uptake. It has been reported that the negatively charged nanotubes had a significant impact on both seed germination and plant growth, especially carbonyl on nanotubes surfaces [40]. According to the published reports, potassium ion channels usually are composed of four subunits of protein, which are symmetric to form a K^+^ selectivity filter for single potassium ion to pass through [41]. The K^+^ selectivity filter consists of amino acid sequence TVGY, and each filter has four binding sites. Each ion-binding site is surrounded by eight oxygen atoms contributed by the backbone carbonyl groups of the amino acid in TVGY sequence and the side-chain of the threonine residue [42]. The carbonyl oxygen atoms from amino acids point to the pore with a diameter of 0.3 nm, which allows K^+^ to pass through, but inhibits Na^+^, Ca^2+^, NH_4_^+^ and other ions going through. It has been confirmed that there was large amounts of carbonyl on the surface of CNPs. Although the CNPs were inhibited to enter BY-2 cells by methyl-β-cyclodextrin and genistein gathering on the outside of membrane, K^+^ accumulation did not decrease compared with the CNPs treated group. In addition, the potassium channel gene *NKT1* was downregulated in the presence of methyl-β-cyclodextrin. Moreover, the CNPs loaded in the nanopore exhibited K^+^ selectivity compared with other cations using the RIC assay. Therefore, the carbonyl may presumably form nanopores similar to potassium channels on the membrane to permit K^+^ penetrating into cells. These results indicated that the CNPs may interact with membrane by simulating biological ion channels for K^+^ uptake, which was another way for K^+^ uptake.

The potential benefits of employing metabolomics have recently been recognized to assess the toxicology of nanomaterials applied in biological system. The effect of CNPs exposure on metabolic profile of amino acid was determined, and 25 kinds of amino acid were analyzed. The amino acid metabolites were upregulated or downregulated in the four exposure groups compared with the control group. CNPs reduced the levels of l-Tyrosine and boosted contents of amino acid, such as l-aspartic acid, l-serine, and l-proline with the increasing concentration of CNPs in cells. The proline content was influenced by the potassium levels to a large extent. Generally, as potassium levels increased, the production of proline showed to rise [43]. It has been reported that the downregulation of amino acid metabolism was associated with the enhancement of oxidative stress [6]. With upregulating some amino acid, especially l-proline, the oxidative stress appeared to be alleviated in the presence of CNPs. These results were consistent with previous publication that the production of ROS was lower under the condition of higher concentration of CNPs.

## Conclusions

In summary, we have revealed that K^+^ accumulation in cells was related to cell growth induced by CNPs and CNPs upregulated gene expression in BY-2 cells, including *NKT1*, *NHA1* and *NtHAK1*. K^+^ accumulation reached the maximum value when the concentration of CNPs was 61.25 μg/mL. The K^+^ accumulation increased with the addition of exogenous K^+^ (≥ 20 mM) in the presence of CNPs. As well, the carboxyl groups on the surface of CNPs were crucial for improving K^+^ uptake. In addition, CNPs may simulate ion channels for K^+^ permeating into cells. These results further revealed mechanism that CNPs promoted growth of plant cells. Comprehensive investigation of the mechanism will facilitate understanding the impact of carbon based nanomaterials exerted on plant cells growth.

## Supplementary information

**Additional file 1: Figure S1.** Effect of exogenous K^+^ on the intracellular K^+^ content in the presence of CNPs in BY-2 cells. A: without addition of exogenous K^+^, B: with addition of exogenous K^+^ at concentration of 20 mM except for control group. Error bars indicate the standard error of the mean (n = 4). Asterisk (*) indicates significant difference compared with control group and group containing 20 mM K^+^ without CNPs (p < 0.05). **Figure S2.** Changes in extracellular matrix pH value treated with different concentration of CNPs. Asterisk (*) indicates significant difference compared with control group and group containing 46.88 mM K^+^ without CNPs (p < 0.05). **Table S1.** Primer sequences.

## Data Availability

All data generated or analyzed during this study are included in this published article and its additional files.

## Abbreviations

CNPs: carbon nanoparticles
MWCNTs: multi-walled carbon nanotubes
GO: graphene oxide
SWCNTs: single-walled carbon nanotubes
NMT: non-invasive micro-test technology
TGA: thermogravimetric analysis
FTIR: fourier transform infrared spectrum
