# Supplementary material for: Carbon nanoparticles enhance potassium uptake via upregulating potassium channel expression and imitating biological ion channels in BY-2 cells
Source: J Nanobiotechnology. 2020 Jan 28;18:21. doi: 10.1186/s12951-020-0581-0 (PMC6986061; doi:10.1186/s12951-020-0581-0)
Supplement: Supplementary file 1 — Additional file 1: Figure S1. Effect of exogenous K+ on the intracellular K+ content in the presence of CNPs in BY-2 cells. A: without addition of exogenous K+, B: with addition of exogenous K+ at concentration of 20 mM except for control group. Error bars indicate the standard error of the mean (n = 4). Asterisk (*) indicates significant difference compared with control group and group containing 20 mM K+ without CNPs (p < 0.05). Figure S2. Changes in extracellular matrix pH value treated with different concentration of CNPs. Asterisk (*) indicates significant difference compared with control group and group containing 46.88 mM K+ without CNPs (p < 0.05). Table S1. Primer sequences. [file 12951_2020_581_MOESM1_ESM.docx]

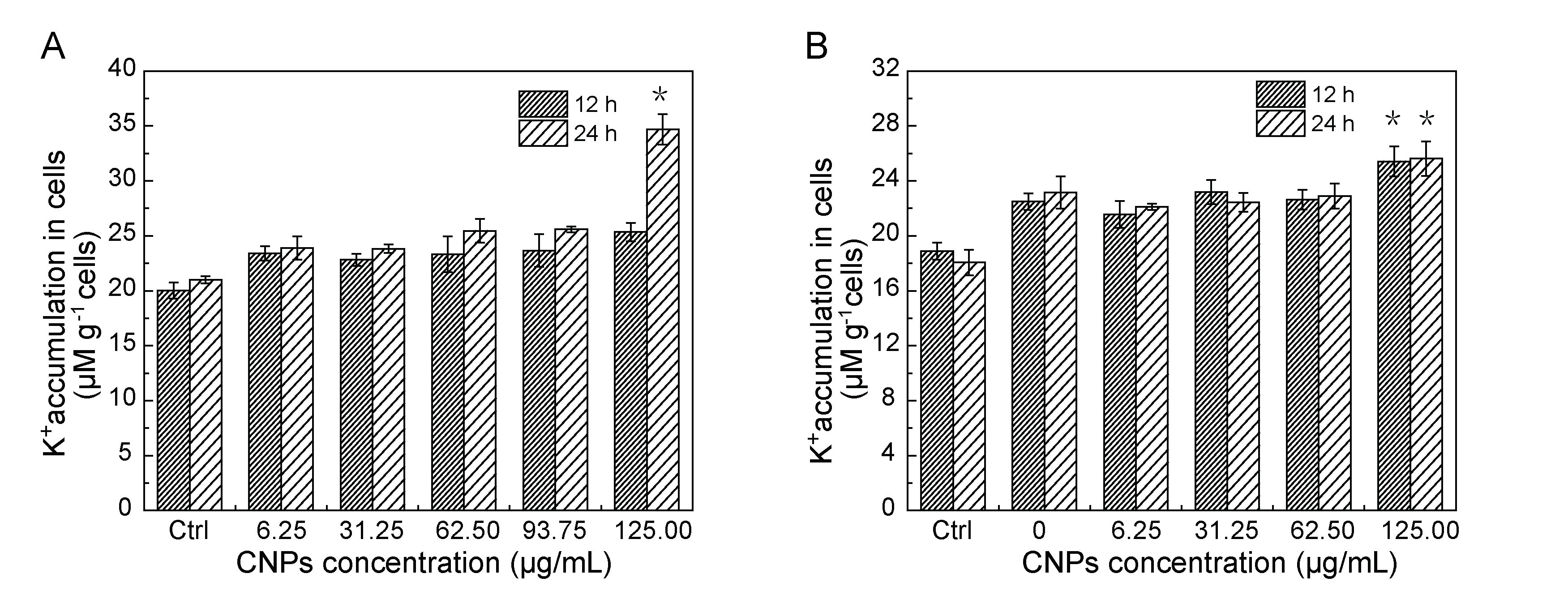


Figure S1. Effect of exogenous K^+^ on the intracellular K^+^ content in the presence of CNPs in BY-2 cells. A: without addition of exogenous K^+^, B: with addition of exogenous K^+^ at concentration of 20 mM except for control group. Error bars indicate the standard error of the mean (n=4). Asterisk (＊) indicates significant difference compared with control group and group containing 20 mM K^+^ without CNPs (p＜0.05).


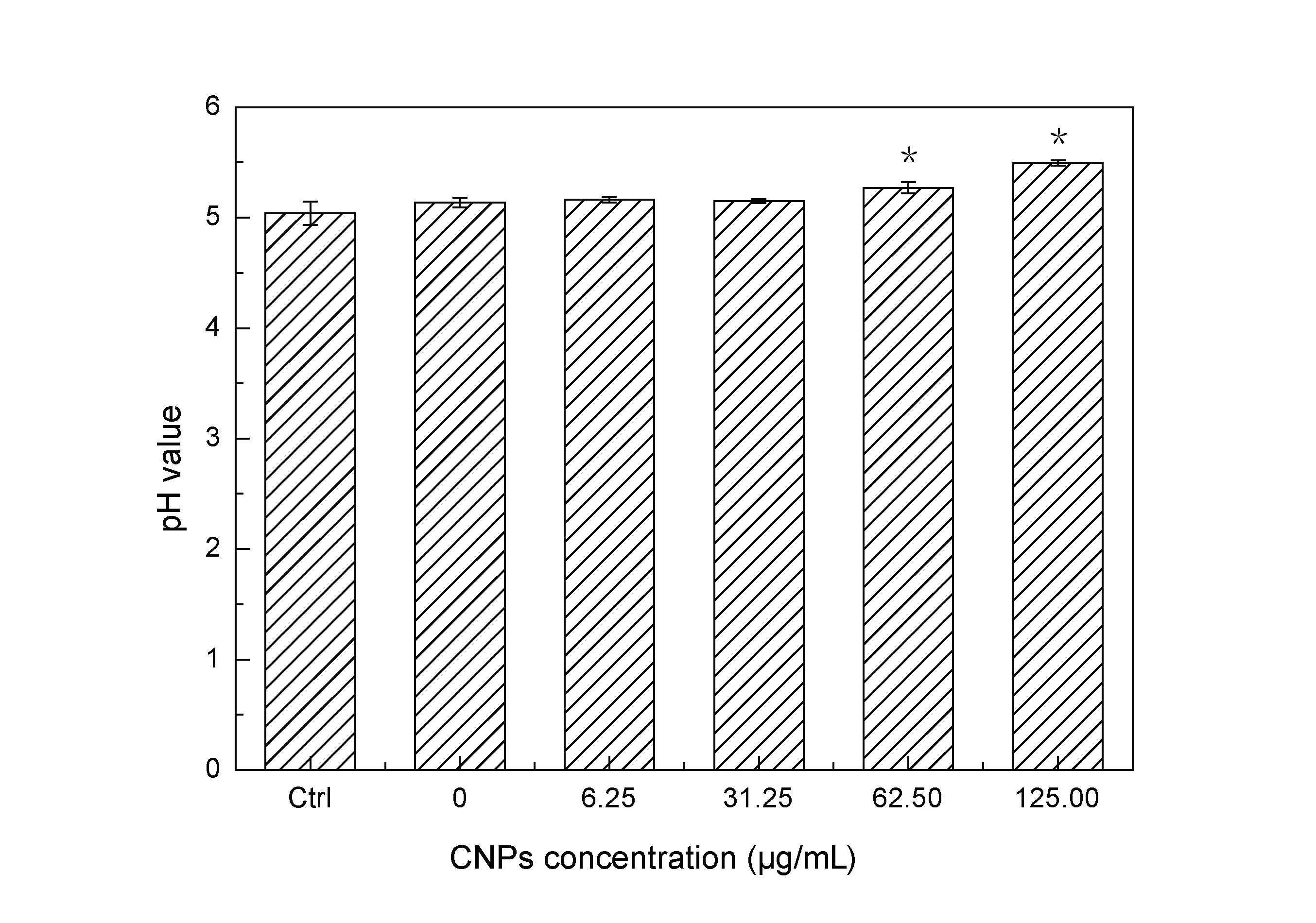


Figure S2. Changes in extracellular matrix pH value treated with different concentration of CNPs. Asterisk (＊) indicates significant difference compared with control group and group containing 46.88 mM K^+^ without CNPs (p＜0.05).

Table S1. Primer sequences.

| Primer | From 5’ to 3’ |
| --- | --- |
| *NKT1* | F:TTGCTGGTGATGGTACTTCAG |
|  | R:TACCCGCCCTAGATTAGTCG |
| *NtKC1* | F:GGTTGACGCCTTCTTTGCTA |
|  | R:GACTGGTCCTCGGTTCATTTT |
| *NTORK1* | F:AGTGAAACAACTTGAGAGTACCTC |
|  | R: GAGAAGCATAAACTGCTACAGTGG |
| *NHA1* | F:GCAAGAGCAGGCATCCAAGA |
|  | R: CCACAGCCAAGGAACGAAGA |
| *NtHAK1* | F:AGTTGGCATTACGGCACCC |
|  | R: GAATTCCTGAAACAAGCTCGG |
| *Actin* | F: GAACGGGAAATTGTCCGCGATGTT |
|  | R: ATGGTAATGACCTGCCCATCTGGT |
